# Accidental Subclavian Artery Catheterization After Central Venous Catheter Placement

**DOI:** 10.7759/cureus.72492

**Published:** 2024-10-27

**Authors:** Sonal Kumar, George Tadros, Otto Montero, Alberto Lopez, Morris Sasson

**Affiliations:** 1 Vascular Surgery, Ross University School of Medicine, Miramar, USA; 2 Vascular Surgery, Cleveland Clinic Florida, Weston, USA

**Keywords:** arterial puncture, catheter-related trauma, central line complications, central venous catheter, iatrogenic arterial injury, subclavian artery injury, subclavian catheterization, ultrasound-guided catheterization, vascular access complications, vascular injury

## Abstract

While central venous catheterization (CVC) is used extensively and for a spectrum of medical indications, including dialysis access, the procedure is not without risks. Addressing and acknowledging potential complications is informative, as such insight can improve patient outcomes and establish clinical guidelines. We present the case of a 34-year-old male with dermatomyositis who sustained a subclavian artery injury in the intensive care unit following internal jugular vein catheterization. This case raises awareness of this complication to enable us to promptly identify and manage similar patients in the future, as well as emphasize refining the procedural technique of physicians at all levels. Due to the rarity of CVC vessel injury, literature describing the consequences is scarce. We discuss teamwork and safety culture, emphasizing how interventions can reduce error risk by improving team collaboration, leader responsiveness, and above all patient outcomes.

## Introduction

Central venous catheterization (CVC) is a foundational procedure used commonly in medical settings and across specialties for varied indications, including the administration of medications, performing dialysis, and observing hemodynamic status. Despite its routine use, CVC placement is not without complications and has the potential to jeopardize patient safety and outcomes. Recent research has addressed some of the central issues associated with CVC. 

Despite advancing infection control protocols, catheter-related bloodstream infections (CRBSIs) remain a pressing concern for CVC [1]. More than five million central venous catheterizations are performed annually in the United States, with over 15% of these procedures expected to result in complications [2-6]. At least 400,000 episodes of vascular-catheter-related bloodstream infections (CRBSI) occur in the USA per year [2]. Thrombotic events following CVC, such as deep vein thrombosis and thrombophlebitis, have also been frequently observed and reported in the literature [3]. Additionally, mechanical complications from CVC such as pneumothorax, hemothorax, and incorrect catheter positioning frequently arise from procedural mistakes or anatomical variants [4,7]. While enhanced ultrasound guidance focuses on reducing CVC complications, consistent monitoring, and prompt intervention are of great importance [5,7]. Lastly, residents or young physicians doing CVCs are undoubtedly more prone to procedural errors, so direct supervision by a qualified physician has the potential to reduce the complication rate by 50% [8].

This article contributes to the deficiency of literature on this topic and provides recommendations to decrease complication rates. Understanding the likelihood of injury is imperative for physicians at all levels of training to improve patient safety and uphold quality patient-focused care.

## Case presentation

A 34-year-old White male with newly diagnosed dermatomyositis presented to the emergency department with chest tightness, shortness of breath, and a dry, non-productive cough. 

Pulmonary medicine was consulted, and a CT chest confirmed extensive, patchy ground glass opacities bilaterally suggestive of interstitial lung disease flare and an acute exacerbation of idiopathic pulmonary fibrosis. The patient was admitted to the medical intensive care unit secondary to acute hypoxemic respiratory failure in the setting of dermatomyositis with worsening oxygen requirements. The patient had no prior history of vascular procedures or traumatic injuries. 

**Figure 1:**
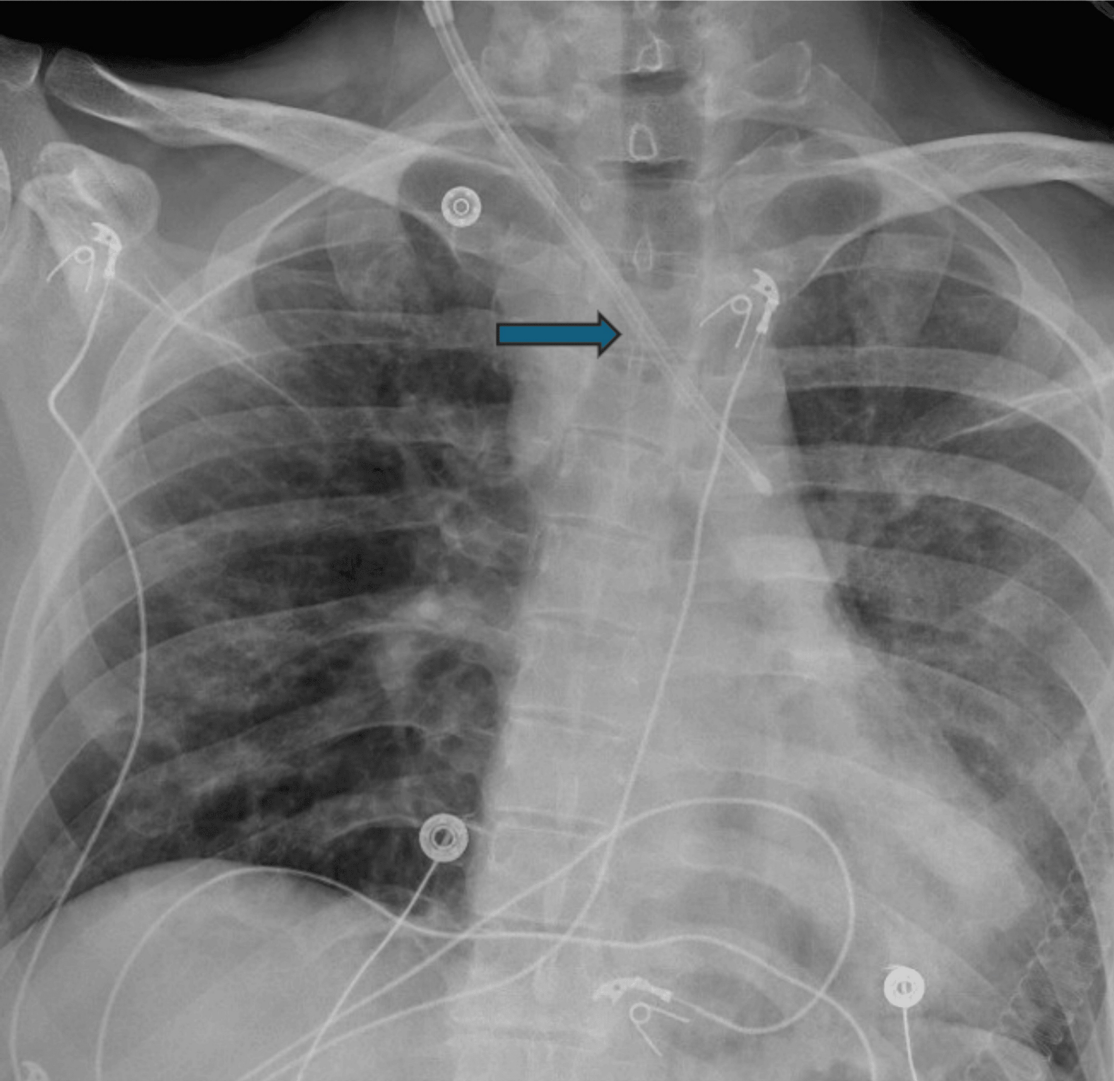
A chest AP radiograph showed a central venous catheter placement in the subclavian artery with its tip ending in the aortic arch (arrow). AP: anterior posterior

**Figure 2:**
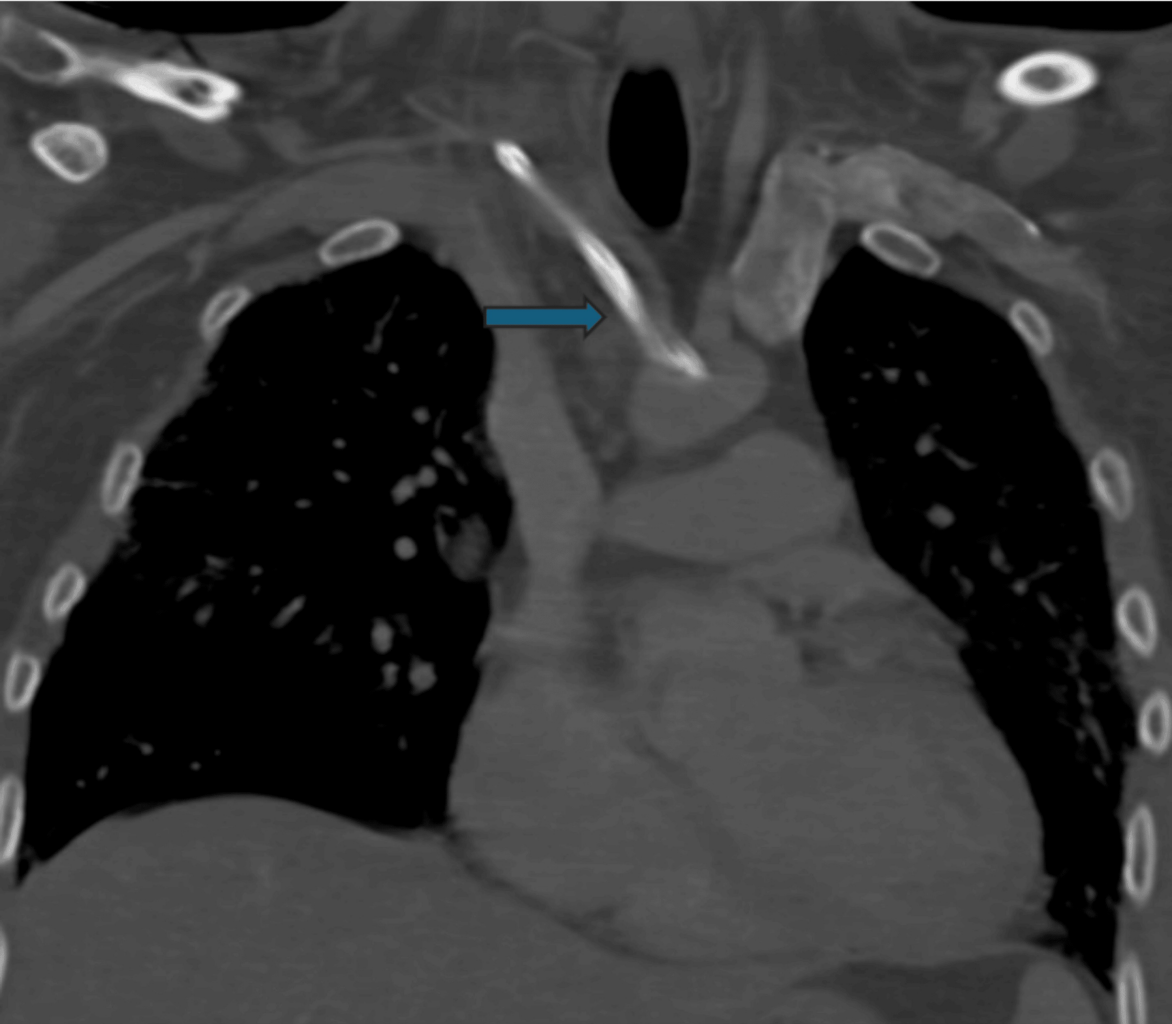
Computed tomography angiography (CTA) head/neck confirmed arterial placement of the central line (arrow).

Upon admission, he underwent placement of the right neck 13F central venous catheter for plasmapheresis, which was needed for treatment. A chest X-ray was ordered per protocol to confirm line placement, and it showed the catheter to be coursing to the left over the aortopulmonary window region, concerning catheter misplacement and possible arterial injury (Figure 1). At this time, vascular surgery was consulted, and computed tomography angiography (CTA) of the head/neck was conducted, which confirmed arterial placement with CVC in the right subclavian artery (Figure 2). The CTA chest was also ordered and showed the arterial placement of the right neck central line with its tip within the distal aortic arch.

Removal of the catheter and repair of the vessel were recommended and discussed with the family and patient. The risk of bleeding, infection, and the potential need for sternotomy were discussed. Vascular surgery was consulted eight days after admission to the hospital; surgery was performed the same day as the consultation, approximately three hours later. Anticoagulation was not initiated. The patient consented to remove the line in the operating room (OR). 

In the OR, the patient was laid supine, placed under general anesthesia, and intubated. The patient was prepped in the usual sterile fashion and draped with exposure from the chin to the knees. The cardiothoracic surgery (CTS) team was notified and was on standby in the event that subclavian artery control was not successful via the supraclavicular incision and a median sternotomy was needed for safe repair. A shoulder roll was placed, and the head turned to the contralateral side. The ipsilateral neck and chest were prepped and draped in the usual sterile fashion. A transverse incision just above the right clavicle was made and carried down the level of the sternocleidomastoid muscle. The sternocleidomastoid muscle heads were divided, and the tissues were carefully dissected around the catheter. The internal jugular vein (IJV) was visualized, and the catheter was noted to have gone through the lateral wall of the IJV and into the right subclavian artery. The carotid was intact and uninjured, not found at the site of the arterial catheter. The catheter was noted to enter and exit the internal jugular vein. The right IJV was repaired with 5-0 Prolene sutures in a running fashion. The subclavian artery was dissected further for enhanced exposure. Dissection was difficult due to the position of the subclavian artery injury, which was deep and posterior to the clavicle. In our scenario, proximal control was not possible due to the very proximal location of the needle entrance point of the right subclavian. This is why median sternotomy was considered, but in the end not required. A “purse string” 4-0 pledged Prolene suture was placed around the catheter, which was then removed and the suture was tied securely. The arteriotomy repair achieved effective hemostasis. Gelfoam thrombin was applied to achieve hemostasis. The platysma was closed with a 3-0 Vicryl suture, and the skin was closed with a running 4-0 Monocryl suture. The skin was dressed with glue and sterile dressing. The patient was awoken from anesthesia, was neurologically intact, and was taken to recovery in stable condition. The patient was extubated with an intact right upper extremity motor and sensory function. The patient’s right brachial and ulnar pulses were palpable. Following the surgery, the patient was transferred to the ICU in stable condition. The rest of the patient’s care was coordinated by the primary team. 

## Discussion

This case discusses one complication of CVC placement, iatrogenic subclavian artery injury, and the steps taken to manage the complication and treat the patient. While CVC is a routine procedure, complications, such as the one sustained by our patient, can lead to complications if not identified and addressed in a timely fashion. The injury in this case demonstrates the need for precise technique and increases awareness during catheter placement, regardless of the medical specialty or training level of the physician. 

Recent literature suggests that complications can be significantly reduced with improved procedural practices. Stringent infection control policies are paramount to prevent such related complications [1]. In addition, with advances in imaging technologies, like real-time ultrasound guidance, mechanical complications can be minimized, which will improve the accuracy of catheter placement [2,7]. Similarly, thrombosis, along with other complications, is less likely to occur if the clinicians are well-trained and follow updated procedural protocols [3].

Continued education and the application of imaging techniques can hugely improve procedural outcomes [4,7]. In this case, immediate surgical intervention was imperative in the treatment of the injury; thus, it is indicative of prompt, efficacious methods of treatment. 

Percutaneous closure devices or covered stents are options for iatrogenic arterial catheter placement, but in this case, the large-bore 13F catheter and proximal subclavian artery injury made them high-risk. Inadequate sealing could have led to significant hemorrhage [8], as proximal control was not feasible. Given the patient's young age, an open repair was preferred to avoid long-term stent complications.

We would also like to emphasize the discussion in the most recent report about procedural training for young physicians and trainees [9]. We believe it is no longer a luxury but an urgent need to avoid post-CVC complications and patient harm. Resident physicians are responsible for their level of skill. Trainees ought to seek support and mentorship and must voice concerns in cases where they are uncomfortable performing this or any other procedure. 

While teamwork is the foundation of safe medical care, it can be undermined by hierarchy-based barriers. In teaching hospitals where supervising physicians evaluate junior learners, trainees may prioritize acquiescence and demonstrate boldness over caution and patient safety. Team dynamics can be improved through teamwork training, including communication and mentorship, and shared prioritization of patient safety goals. Rewarding team members who identify errors and safety risks is an example of a potential intervention that would improve comfort in expressing safety-related concerns and improve leadership responsiveness.

## Conclusions

Our case describes one of the possible and significant complications that could arise from an iatrogenic injury to the subclavian artery during CVC placement. Iatrogenic vascular injury is a consequence of poor technique, lack of ultrasound use, inexperience, and inadequate procedural oversight. Our case exemplifies the importance of early detection and timely management of CVC injury. 
